# Pancreatic Islet Transcriptional Enhancers and Diabetes

**DOI:** 10.1007/s11892-019-1230-6

**Published:** 2019-11-21

**Authors:** Inês Cebola

**Affiliations:** Section of Genetics and Genomics, Department of Metabolism, Digestion and Reproduction, Imperial College London, Hammersmith Campus, ICTEM 5th floor, Du Cane Road, London, W12 0NN UK

**Keywords:** Transcriptional enhancers, Human genetics, Type 2 diabetes, Gene regulation, Epigenomics, Noncoding genome function

## Abstract

**Purpose of Review:**

Common genetic variants that associate with type 2 diabetes risk are markedly enriched in pancreatic islet transcriptional enhancers. This review discusses current advances in the annotation of islet enhancer variants and their target genes.

**Recent Findings:**

Recent methodological advances now allow genetic and functional mapping of diabetes causal variants at unprecedented resolution. Mapping of enhancer-promoter interactions in human islets has provided a unique appreciation of the complexity of islet gene regulatory processes and enabled direct association of noncoding diabetes risk variants to their target genes.

**Summary:**

The recently improved human islet enhancer annotations constitute a framework for the interpretation of diabetes genetic signals in the context of pancreatic islet gene regulation. In the future, integration of existing and yet to come regulatory maps with genetic fine-mapping efforts and in-depth functional characterization will foster the discovery of novel diabetes molecular risk mechanisms.

## Introduction

In recent years, clinical genomics and the investigation of noncoding genome functions, including microRNAs (miRNAs), long noncoding RNAs (lncRNAs) and transcriptional enhancers, have proved to be fertile ground to the discovery of genetic disease mechanisms [1]. Sequence variation leading to altered enhancer function is now appreciated as a driving mechanism in cancer [2–4] and a number of Mendelian disorders are caused by mutations affecting tissue-specific *cis*-regulatory elements [5], including non-syndromic pancreas agenesis [6]. The overwhelming majority of genetic variants associated with common human diseases and disease-associated traits are noncoding and concentrate within accessible chromatin regions of disease-relevant tissues [7]. This observation is also true for type 2 diabetes (T2D), for which common noncoding variants predominantly influence risk [8•], residing in pancreatic islet transcriptional enhancers [9–12, 13••, 14, 15], particularly within clustered enhancers [9–13]. Thus, most of the genetic susceptibility to T2D seems to arise from islet regulatory defects, which aligns well with the observation that many T2D-associated variants also affect measures of pancreatic β cell function [14–16]. Moreover, recent reports indicate that islet enhancers also harbour genetic variants that confer susceptibility to type 1 diabetes [17], even if not to the same extent as for T2D [18]. These observations combined with recent technological advances have prompted the production of evermore refined maps of human islet *cis*-regulatory elements in recent years, including whole islet, cell type-specific and disease state-specific maps, improving the functional annotation of diabetes risk variants. In this review, I cover the progress that has been made in the mapping of active islet enhancers and its implications for the identification of causal diabetes risk variants.

## A Decade of Islet Regulomes: Restricting the Search Space for Diabetes Causal Variants

Since T2D risk variants are mostly noncoding [8•] and predominantly enriched in islet enhancers [9–12, 13••, 14, 15], defining the regulatory regions that are active in pancreatic islets is key to identifying truly causal variants. Several iterations of human islet *cis*-regulatory maps or *regulomes* have been built to date and a number of publicly accessible genome browsers now host human islet regulomes, including the Roadmap Epigenomics Project (https://egg2.wustl.edu/roadmap/web_portal/), the Islet Regulome Browser (www.isletregulome.org/) and the Diabetes Epigenome Atlas (https://www.t2depigenome.org/).

Although providing an exhaustive overview of the evolution of islet enhancer maps is not the aim of this review, a few general points are still worth mentioning. In earlier maps, islet enhancers were defined as broad domains enriched in typical active chromatin marks (H3K4me1 and H3K27ac), often spanning several kilobases, whereas currently enhancers are more commonly defined as accessible chromatin sites typically spanning 300–500 bp that overlap chromatin regions enriched in active chromatin features. Moreover, due to lower starting material requirements, ATAC-seq [19] was adopted in recent years as the standard method to map regulatory chromatin sites in human islets [12, 13••, 20, 21•, 22].

Improved understanding of the biochemical properties that distinguish active from inactive chromatin has enabled a progressive subcategorization of islet accessible chromatin regions and identification of specific enhancer subsets that are more relevant for diabetes risk. For example, Miguel-Escalada et al. have recently sub-classified active human islet enhancers into three classes according to their enrichment in active chromatin features (H3K27ac and the coactivator complex Mediator) [13••]. This subclassification revealed a subset of 13,635 strong islet enhancers that tended to regulate islet-specific genes and contribute more to diabetes and β cell-related trait heritability than other enhancer subsets [13••]. In another study, profiling of DNA methylation in human islets from 10 donors using whole-genome bisulphite sequencing revealed that T2D risk variants concentrate within open and hypomethylated regions [21•]. Future studies profiling additional enhancer features, including eRNA production [23] and enrichment in additional enhancer-associated histone modifications [24], have the potential to help obtain further granularity to recognize additional diabetes-relevant islet enhancer categories.

## Strategies to Improve Causal Variant Mapping

A big challenge to transpose the knowledge arising from genetic association studies to disease mechanisms is the fact that the most significant variants identified in genome-wide association studies (GWAS) may not be the true causal ones, but simply be in high linkage disequilibrium with the causal variant. Several approaches may be taken to improve the resolution of causal variant mapping, including the analysis of larger numbers of individuals [25••] in diverse populations [26–29], and by applying genetic fine-mapping [18, 25••, 30–32] (reviewed in [33]). Remarkably, a recent meta-analysis of ~ 900,000 European descent individuals using genetic fine-mapping has expanded the repertoire of T2D risk loci to > 240 and isolated single causal variants at 18 association signals [25••]. Parallel efforts with > 400,000 East-Asian individuals implicated 56 additional loci, highlighting that the quest to identify T2D-associated loci is probably not over yet [34••].

**Fig. 1:**
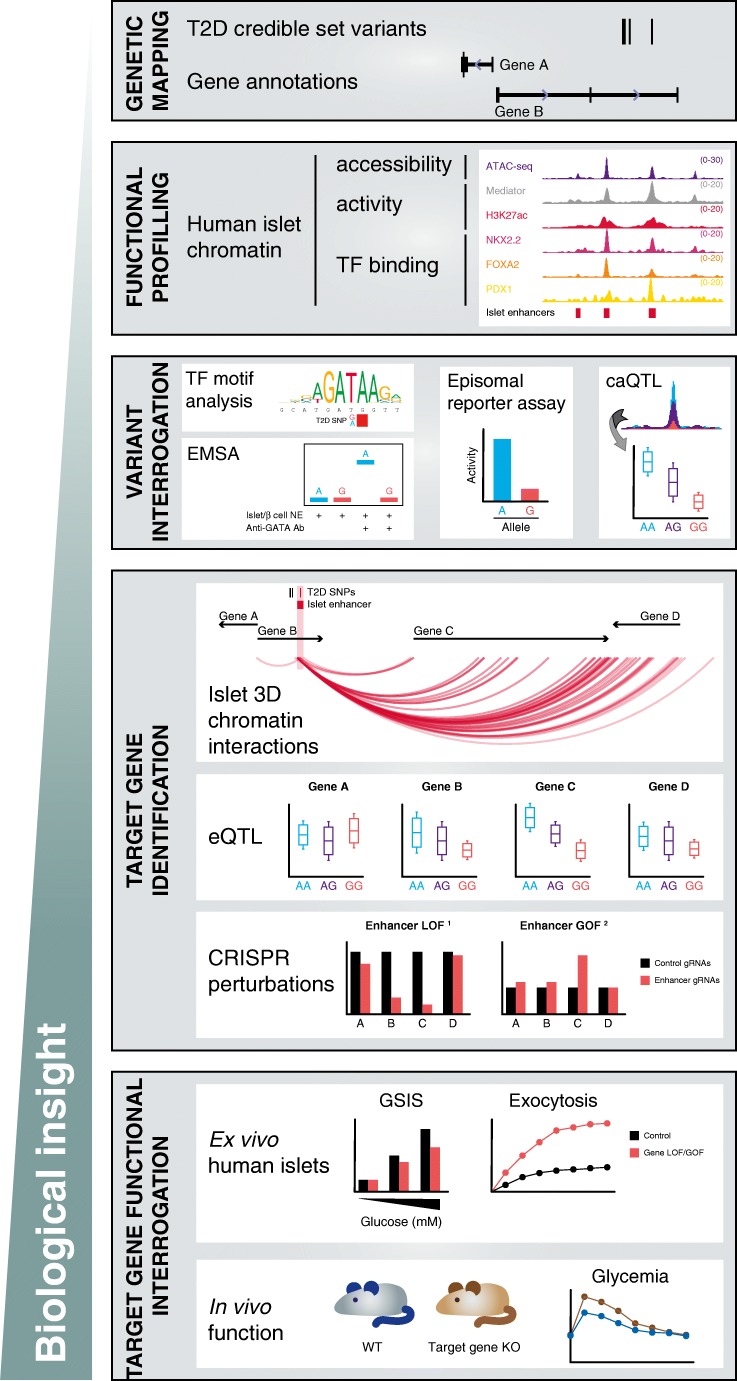
Overview of the workflow to prioritize noncoding variants and target gene investigation in T2D. Enhancer loss-of-function (LOF)^1^: enhancer LOF can be achieved by either indels at the core region (transcription factor binding sites), full deletion, or CRISPR-mediated inhibition (CRISPRi). Enhancer gain-of-function (GOF)^2^: CRISPR-mediated activation. Please note that the schematic does not provide an exhaustive list of all possible methods to prioritize genome-wide association study (GWAS) variants due to space limitations, providing instead the most frequently applied approaches

It is reasonable to assume that for a given T2D locus where coding variants are not likely to be causal, variants residing within *cis*-regulatory elements are more likely to be driving the genetic risk (Fig. 1). This principle is the basis of FGWAS [35], in which the likelihood of a given GWAS variant being causal is weighted by functional annotations such as histone modification enrichment, chromatin accessibility or DNA methylation status [21•]. FGWAS has already been applied by a number of studies to fine-map T2D risk loci [8•, 21•, 25••, 36], with reported reductions in the order of 35% of the number of T2D-associated variants at specific loci [8•].

In order to correctly refine the sets of T2D variants using functional genomic data, not only sample purity and data quality are important, but also the type of functional data that is used to prioritize variants. For example, it has been observed that islet chromatin accessibility is more predictive of regulatory impact than DNA methylation [21•]. Moreover, clustered enhancers are more enriched in T2D variants than “orphan” enhancers [9, 10]. More recent analyses have also demonstrated that refining islet enhancer maps can help identify a restricted segment of the genome that is more relevant for islet gene regulation and, consequently, to T2D risk via β cell function impairment [13••]. In this study, an enhancer subset defined by strong enhancers that cluster in 3D (see the section “Enhancer Clusters in 3D” for details) was found to contribute more to the heritability of T2D and traits related of β cell function (HOMA-B and insulinogenic index from oral glucose tolerance test) compared to other classes of enhancers. Notably, this result was achieved not by covering a larger proportion of the genome with this annotation but, on the contrary, by restricting the annotation to open chromatin sites (marked by ATAC-seq) within regions that presented multiple features associated with tissue-specific regulation, such as stronger ChIP-seq enrichment in H3K27ac/Mediator, and being promoter-interacting.

## Demonstrating Variant Causality

It is assumed that genetic variation in islet enhancers leads to changes in transcription factor binding and, consequently, enhancer activity and gene regulation. One way to measure this is by computing changes in transcription factor (TF) binding affinity using TF motif analysis, but this strategy is limited to known TF recognition sequences, which have only been identified for two thirds of human TFs [37]. Moreover, the presence of a particular TF motif in an enhancer does not necessarily reflect that it is a genuine binding site for that TF. In reality, *in vivo* TF binding is influenced by multiple factors, including cofactors, cooperativity, concentration and even chromatin shape [38]. A less biased approach is to directly interrogate sequence-dependent enhancer activity (Fig. 1). This strategy was successfully applied for a number of T2D-associated enhancer variants (see Table 1 for examples). Nonetheless, enhancer reporter assays also have limitations, as they interrogate candidate enhancer sequences outside of their native context. An alternative to address this question is to directly probe the epigenomic profiles from multiple individuals to identify genetic variants that confer allele-dependent chromatin activity (chromatin activity quantitative trait loci, QTLs) (Fig. 1). Three studies have already yielded encouraging results by performing chromatin accessibility QTL (caQTL) analysis on human islet ATAC-seq profiles from 17 [21•], 19 [20] and 23 individuals [22]. In one of these studies, Thurner et al. confidently reduced T2D GWAS signals at *CAMK1D*, *KLHDC5* and *ADCY5* to single regulatory variants by combining genetic fine-mapping, FGWAS and caQTL analysis [21•]. In another study, Khetan et al. identified nearly three thousand SNPs that yielded allele-dependent changes in islet chromatin accessibility, including 13 T2D-associated variants [20]. This number was later increased to 24 T2D variants by addition of four new islet samples [22]. Thus, future analyses with larger sample sizes and meta-analysis of existing human islet ATAC-seq datasets [12, 13, 20, 21•, 22, 39] hold promise for uncovering additional T2D risk variants that influence islet enhancer function. Similarly, allelic imbalance analysis of active chromatin histone modifications, such as H3K27ac, and islet TF occupancy may assist the identification of T2D regulatory variants, as it has been demonstrated at individual T2D loci [17, 40].

**Table 1:** Functional T2D-associated variants in islet enhancers

T2D locus	Variant	Risk allele effect direction^1^	Likely effector transcript(s) in islets^2^	Experimental evidence of variant/enhancer function in islet cells							TF
				Luciferase	EMSA	eQTL	caQTL	hQTL	mQTL	CRISPR	
*ADCY5*	rs11708067	Down	*ADCY5*	[40]	[40]	[40, 57]	[20, 21•]	[40]	[21•]	[40]	
*ARAP1/STARD10*	rs140130268	Down	*STARD10*^3^	[36]		[13••, 36, 57]					
*C2CD4A/B*	rs7163757	Up	*C2CD4A/B*^3^	[98]		[98]				[13••]	NFAT
*CDC123/CAMK1D*	rs11257655	Up	*CAMK1D* and *OPTN*	[13••, 99]	[99]	[13••, 57]	[21•]			[13••]	FOXA1/FOXA2
*GLIS3*	rs4237150^4^	Up	*GLIS3*	[13••] [17]			[17]	[17]		[13••]	
*IL20RA*	rs6937795	Up	*IL20RA*	[20]			[20]				
*DGKB*	rs10228796^4^	Up	*DGKB*	[59•]	[59•]	[59•]					
*JAZF1*	rs1635852	Down	*JAZF1*	[100]	[100]						PDX1
*KLHDC5*	rs10842991	Up	*KLHDC5*^3^			[12]	[21•]				PAX6
*MTNR1B*	rs10830963	Up	*MTNR1B*	[30]	[30]	[57, 101]	[20]				NEUROD1
*TCF7L2*	rs7903146	Up	*TCF7L2*	[102]		[59•]	[102]			[13••]	
*ZBED3*	rs4457054	Up	*PDE8B* and *ZBED3*^3^	[22]		[59•]				[13••]	
*ZFAND3*	rs58692659	Down	*ZFAND3* and *MDGA1*^3^	[10]	[10]					[13••]	NEUROD1
*ZMIZ1*	rs12571751	Up	*ZMIZ1*^3^			[57]	[20]				

^1^Down: risk allele associates with less enhancer activity/target gene mRNA expression in islets; Up: risk allele associates with more enhancer activity/target gene mRNA expression in islets

^2^Only transcripts with the strongest evidence are listed

^3^Additional putative effector transcripts for this locus have been identified by promoter capture Hi-C analysis in [13••]

^4^Other T2D risk variants in the same islet enhancer also alter enhancer activity in episomal reporter assays

## Enhancer Clusters in 3D

Three-dimensional (3D) chromatin studies with techniques such as Hi-C [41] and genome architecture mapping (GAM) [42], which allow identification of pairwise chromatin interactions in the entire genome, have put back in the spotlight 3D genome organization in the context of gene regulation and disease, and a number of recent reviews cover this topic extensively [43–45, 46•].

In one of the earlier maps of islet enhancers, the authors noted that islet-specific genes tended to be regulated not by one, but by multiple enhancer clusters [10]. In the same study, the authors detected long-range interactions between single promoters and multiple enhancers by performing circular chromosome conformation capture coupled with high-throughput sequencing (4C-seq) in nine loci [10]. These observations lend plausibility to the hypothesis that clustered enhancers form higher-order 3D regulatory structures to control cell-specific expression, and agree with well-characterized higher-order 3D regulatory structures detected in specific loci, including the *HoxD* cluster [47], and the α- and β-globin loci [48, 49]. However, the extent to which the formation of higher-order 3D regulatory structures is a general property of enhancers and enhancer clusters, and its contribution to the establishment of tissue-specific *cis*-regulatory programs could not be fully appreciated in these studies.

Recently, Ferrer and colleagues have addressed this question by generating a high-resolution map of promoter-mediated interactions in human pancreatic islets from four different donors using promoter capture Hi-C (pcHi-C) [13••]. In agreement with earlier observations [10], the authors observed that (1) islet genes often interact with multiple enhancers and clusters of enhancers and that (2) islet enhancers often interact with multiple target genes. Similar observations were made in a lower resolution map of 3D chromatin contacts obtained by performing Hi-C in three human islet samples [22]. Overall, this demonstrates that islet enhancers and their target promoters reside in restricted 3D chromatin domains, named *enhancer hubs* for their highly connected nature [13••]. Using a systematic approach, the authors defined > 1300 islet enhancer hubs, defining the portion of the genome that is particularly enriched in islet enhancers and islet enhancer-promoter interactions. Enhancer hubs include genes that tend to be more islet enriched and predominantly involved in islet cell function and diabetes risk [13••]. These results agree with work performed in other human cell lineages, where super-enhancers were found to regulate tissue-specific genes within frequently interacting regions (FIREs) [50]. Although the methods applied in these studies could not discern whether the detected pairwise chromatin interactions occur simultaneously or alternate, multi-way contacts between enhancers and promoters have been detected in other settings using techniques such as GAM [42], Tri-C [48] and multi-contact 4C [51]. It is thus likely that similar multi-way enhancer-promoter contacts exist in human pancreatic islet chromatin.

Beyond their role as genomic regions that passively harbour regulatory elements and genes important for tissue-specific regulation, there is evidence indicating that enhancer hubs also represent functional units that are important for islet gene regulation. Multiple lines of evidence support this notion, both in steady-state and in dynamic settings. First, the activity of enhancers and genes contained in the same hub tends to correlate more than for those located outside hubs [13••], an observation analogous to *cis*-regulatory domains recently defined in a large series of lymphoblastoid cell lines and primary fibroblasts [52]. Second, when comparing enhancer activity in two different glucose concentrations (4 and 11 mM), hub enhancers frequently showed coordinated changes in their activity, not only in terms of direction of effect, but also in effect size [13••]. Third, perturbations of single hub enhancers or promoters with CRISPR in the glucose-responsive pancreatic β cell line EndoC βH3 [53] frequently yielded changes in the expression of multiple genes in the same hub, demonstrating connectivity of hub *cis*-regulatory elements [13••]. There is therefore cumulative evidence indicating that 3D regulatory domains such as enhancer hubs are key for the establishment and maintenance of cell identity and function. Given the presence of many T2D risk variants in islet enhancers, particularly in strong hub enhancers [13••], these observations raise the question of whether individual T2D variants could be involved in dysregulation of multiple genes and gene pathways.

## Harnessing 3D Genome Maps to Identify Diabetes Effector Transcripts

On its own, the observation that T2D risk variants predominantly locate to islet enhancers, will not bring us closer to identifying *disease effector* transcripts, as genome folding enables transcriptional enhancers to act over genes at varying distances in the linear genome, sometimes even over non-adjacent genes located hundreds of kilobases away (reviewed in [44]). The top obesity-associated locus illustrates well this model, in which the risk variant resides in an intronic enhancer at *FTO* and regulates the expression of two distal genes (*IRX3* and *IRX5*), but not the *FTO* gene [54, 55•]. Thus, enhancer-promoter functional relationships cannot be by simply inferred by examining linear representations of the genome.

A few studies have aimed to link noncoding T2D risk variants to target genes using human islet expression quantitative trait loci (eQTL) analysis [13••, 56–58, 59•], the latest of which included pancreatic islet RNA-seq from 420 donors [59•] and identified candidate effector transcripts for 23 loci. While the results are encouraging, they still fall short from delivering a comprehensive assignment of regulatory variants to effector transcripts. Approaches such as chromosome conformation capture (3C) techniques, including Hi-C [41], ChIA-PET [60] and HiChIP [61], yield genome-scale maps of long-range functional chromatin interactions, but it should be noted that 3C methods are usually biased for detection of long-range interactions. Thus, eQTL and chromatin interaction studies should be taken as complementary, rather than alternative, approaches to identity disease effector transcripts (Fig. 1).

Islet 3D promoter-enhancer contacts were first used to define the regulatory landscapes for a select number of loci [10, 40, 62]. More recently, two studies aimed to associate islet enhancers with target genes using genome-scale 3D chromatin contact mapping in human pancreatic islets [13••, 22] and one in the pancreatic β cell line EndoC βH1 [63]. Greenwald et al. generated Hi-C maps in three human islet samples, identifying 3022 islet enhancers (6.7% of all enhancers mapped in the study) looping to at least one promoter [22]. As in other reports of enhancer-promoter 3D interactions, islet chromatin maps have revealed substantial number of very long-range interactions, sometimes spanning over 1 Mb [13••, 22]. Hi-C and ChIA-PET in EndoC βH1 cells demonstrated the overall suitability of this cell line to study islet *cis*-regulatory functions, with high correlation of interaction profiles across most loci [63]. Hi-C maps, however, offer limited resolution to identify specific functional interactions [64], and targeted methods that enrich Hi-C libraries for genomic regions of interest have the potential to uncover a larger number of functional 3D chromatin interactions, being therefore more informative on the target genes of disease-associated distal regulatory elements [65]. Following this logic, Miguel-Escalada et al. applied a variant of Hi-C, promoter capture Hi-C (pcHi-C), to enrich the maps of islet 3D chromatin interactions in promoter-mediated interactions [13••]. Using this approach, the authors substantially improved the mapping of enhancer-promoter interactions, detecting one or more interactions for ~ 40% of all annotated enhancers (18,031 enhancers). The highly connected nature of particular loci and the definition of enhancer hubs (see the section “Enhancer Clusters in 3D” for details) allowed the authors to infer target genes for a further 40% of the mapped islet enhancers, assigning in total 53 T2D- or fasting glycaemia-associated enhancers to at least one target gene. Surprisingly though, in 75% of the loci where at least one risk SNP falls in an islet enhancer, the authors linked it to one or more distal genes [13••]. This observation has been partially supported by human islet eQTL studies, where a few T2D-associated SNPs have been linked to genes other than their closest, including variants at *CDC123* (linked to *CAMK1D*), *ARAP1* (*STARD10*) and *ZBED3* (*PDE8B*) [13••, 57, 58, 59•], and by CRISPR-mediated perturbations of diabetes-associated enhancers [13••].

At the *ZBED3* locus, for example, CRISPR-mediated activation or repression of an islet enhancer carrying a regulatory T2D risk variant [22, 25••] in EndoC βH3 cells resulted in changes in the expression of multiple genes [13••]. Of the affected transcripts, *PDE8B* showed the strongest response to the enhancer perturbations. This result is quite interesting in light of the most recent human islet eQTL study, where *PDE8B* was identified as the effector transcript of this association signal [59•]. In this case, *PDE8B* seems like a good candidate to pursue with functional studies, as it encodes for a high-affinity cAMP-specific phosphodiesterase and its reduction has been proposed as an approach to enhance insulin response to glucose [66]. It must be noted however that changes in *PDE8B* levels may not make up the full story in this T2D locus, as the CRISPR enhancer perturbations in human β cells affected additional genes, including the lncRNA *ZBED3-AS1* and *ZBED3* itself [13••]. Long noncoding RNAs can have diverse functions and have been proposed as targets for modulation of diabetes-relevant genes in islets [67]. ZBED3 is a secreted protein, whose higher circulating levels have been associated with insulin resistance [68] and metabolic syndrome [69]. Furthermore, this T2D-associated enhancer is also active in adipose tissue [70]. Thus, genetic variation at *ZBED3* could in theory contribute to diabetes risk via multiple cell-specific processes. Overall, these results illustrate how complex the genetic factors that contribute to T2D may be from a molecular standpoint, and exemplify the complementarity of islet 3D genome maps, eQTL and (epi)genome editing approaches to better define disease effector transcripts and gain insights into the molecular underpinnings of specific GWAS signals. More generally, these studies have important implications for the general interpretation of GWAS signals, which traditionally assign association signals to their nearest gene.

## Islet Enhancer Landscapes Are Dynamic

The interaction between chromatin landscapes and T2D goes beyond the co-localization of risk variants with islet enhancers. In reality, islet enhancers are dynamically regulated, responding to external stimuli such as elevated glucose concentrations [13••] or exposure to pro-inflammatory cytokines [71••]. Thus, mapping of islet enhancers under different conditions may uncover new diabetes-related molecular mechanisms, as it has been recently shown in the context of type 1 diabetes [71••].

Studies comparing islets from donors with diabetes versus those from donors without the disease have demonstrated that T2D associates with changes at both transcriptional [56, 72] (single-cell studies addressing this question have been reviewed in [73•]) and chromatin levels [20, 21•, 39, 74, 75]. Significant changes were already detected when comparing the ATAC-seq profiles of islets from five donors with diabetes versus five donors without diabetes [20]. It must be noted however that the majority of T2D-associated changes were quantitative, not qualitative. In other words, the authors did not find evidence that T2D islets have a different set of active enhancers. Instead, the activity level of existing enhancers was found altered [20]. This observation seems sensible considering that T2D is progressive in nature and does not constitute a severe disease with strong transcriptional phenotypes, as observed in developmental disorders or cancer.

Further supporting the dynamic nature of islet chromatin landscapes, global loss of polycomb repression has been associated with β cell dedifferentiation, diabetes and age [74, 76], while other studies identified differentially methylated regions in T2D islets [21•, 75]. Altogether, these observations render several questions that should be addressed in future studies: do T2D risk variants affect islet enhancer dynamics, and do factors such as age and metabolic state change the regulatory impact of T2D variants?

## Islet Cell-Specific Enhancers

As shown in the sections above, the study of whole human islets has provided important insights into the genetic architecture of T2D, fine-mapping noncoding sequences that are important for islet gene regulation. However, these studies were not designed to address the cellular heterogeneity of pancreatic islets [77], in which distinct endocrine cell lineages contribute to glucose homeostasis, including the glucagon-producing α, insulin-producing β and somatostatin-producing δ cells. Islet morphological and functional heterogeneity goes beyond the known endocrine cell lineages, as different β cell subpopulations have also been detected [78–80]. Furthermore, analysis of whole islets may not detect features restricted to rare, but nonetheless important, endocrine cell types. Reflecting the functional heterogeneity of islet cells, their transcriptomes differ substantially, as it has been observed in sorted cell populations [81], by deploying single-cell RNA-seq [73•] and, more recently, using single-molecule RNA FISH [82]. Transcriptional-level differences often reflect different chromatin landscapes. Indeed, even closely related cell lineages, such as pancreatic islet α and β cells, show differences at chromatin level, with differential methylation of cell-specific enhancer sites [83]. In an elegant study by Seung Kim and colleagues, the authors combined purification of specific pancreatic cell populations with ATAC-seq and ChIP-seq analysis to generate regulomes for human α, β, acinar and ductal cells [84••], identifying thousands of enhancers and promoters that are lineage-specific, including 3999 β and 5316 α cell-specific regulatory regions. In these cell-specific regulatory maps, endocrine lineage-specific regions tended to locate near genes generally involved in glucose homeostasis (e.g. *INS*, *GCG*, *GLP1R*, *IRS1/2*) and were enriched in T2D-associated variants, in contrast with non-endocrine lineages [84••].

More recently, single nuclei ATAC-seq (snATAC-seq) was deployed on human islets from three donors, revealing 13 cell clusters with different regulatory landscapes, which included the classic hormone expressing α, β and δ cells, but also usually less appreciated islet cell populations such as immune and endothelial cells [85••]. Reflecting the different regulatory landscapes that co-occur in islets, this study revealed that regulatory elements of different lineages are enriched in different sets of TF recognition sequences. Moreover, in agreement with reports of heterogeneity among β cells [78–80], the authors detected two β cell clusters characterized by different accessibility of the insulin promoter (INS^high^ and INS^low^). Interestingly, although both INS^high^ and INS^low^ accessible sites were enriched in T2D- and fasting glycemia-associated variants, INS^high^ sites revealed a stronger enrichment [85••]. The value of these cell-specific regulatory maps is exemplified at *DGKB*, where a fine-mapped T2D risk variant that modulates regulatory activity [59•] was found in an INS^high^-specific accessible region [85••]. Strikingly, this regulatory region was not previously annotated as active in bulk islet enhancer maps [59•]. Altogether, these studies provide a new layer of detail for the interpretation of T2D-associated variants and will aid the identification of affected cell-specific processes.

## Perspectives and Future Directions

Great progress has been made in recent years to define the epigenomic landscape of human pancreatic islets, but the studies discussed in this review also highlight that the picture we currently have of the genetic architecture of polygenic diabetes is probably a lot more complex than previously anticipated. Future studies are likely to uncover even more common variants that confer diabetes risk. In particular, the greater investigation of non-European populations is likely to point to novel implicated loci [34••].

On the other hand, the advent of technologies that enable epigenomic profiling at single-cell resolution is expected to further advance the identification of cell-specific, as well as developmental-, metabolic- and disease stage-specific regulatory elements. A recently developed strategy proposed by the Buenrostro lab is particularly promising, having taken the throughput of single-cell ATAC-seq to the hundreds of thousands of cells with deeper genome coverage than previous methods [86]. Similar progress is being made to profile chromatin histone modifications at single-cell level, with single-cell ChIP-seq [87] and CUT&Tag [88]. It is therefore expected that in the near future more studies will address the regulatory landscapes of human pancreatic islets at single-cell level, hopefully obtaining important insights into the *cis*-regulatory networks that drive islet dysfunction in diabetes, as demonstrated by the first islet epigenomic maps with single-cell resolution [85••]. Greater attention is also expected to be given to the regulatory mechanisms that associate with β cell dysfunction during different diabetes disease trajectories [89•, 90].

In order to draw clinically relevant conclusions, genomic evidence of causality, including genetic association, epigenomic annotation, eQTL and caQTL, will have to pass through detailed functional validation in appropriate cellular and animal models (Fig. 1). Mechanistic studies of T2D-associated enhancers and putative effector transcripts should be designed taking into account the direction of effect of T2D variants, as not all T2D risk variants lead to loss-of-function (Table 1). Furthermore, future examination of risk variants should aim to go beyond regulatory and transcriptional outputs and also assess relevant cellular functions, as previously shown at *ZMIZ1* [57], *ADCY5* [40], *ARAP1/STARD10* [36], and *SLC30A8* [91] and at genome-scale [92]. β cell genome and epigenome editing at individual loci [13••, 40] and in the shape of large-scale screens [93] have already started to provide a better understanding of the regulatory mechanisms that operate at T2D-associated loci. It is thus expected that additional application of these methodologies to investigate putative causal variants in their genomic and cellular contexts will advance our understanding of diabetes genetic risk processes. Balboa et al. have recently provided a thorough overview of the applications of genome editing in human pancreatic β cell models [94••].

Despite the strong enrichment of T2D variants in islet enhancers, it is clear that not all risk variants act via islet dysfunction, as some correlate with insulin resistance [16, 95]. What is more, a subset of T2D-associated islet enhancers are also active in other diabetes-relevant tissues, such as adipose tissue and the liver [25••]. Thus, combining islet epigenomic maps with those of other relevant tissues will enable a more comprehensive characterization of risk variants.

Altogether, these studies have the potential to enable stratification of individuals by genetic risk of undergoing a specific disease trajectory [89•, 96]. At least five distinct pathways have been reported to drive T2D risk, including two related to β cell function [97•]. This concept has been recently tested by deploying islet enhancer maps to identify individuals at higher risk of developing T2D due to islet dysfunction [13••]. Individuals in this group tended to develop T2D at a younger age and with lower BMI, resembling to some extent individuals who present monogenic forms of diabetes, which is predominantly caused by mutations in islet TFs. Future studies with inclusion of even more refined enhancer maps for islets and other T2D-relevant tissues may therefore help deliver personalized medicine to at least a subset of patients with T2D.

## Conclusions

Great progress has been made in recent years to assist efforts to define islet cell-specific diabetes risk mechanisms using enhancer maps. Nevertheless, the studies discussed in this review also highlight that the picture we currently have of the genetic architecture of polygenic diabetes is probably still incomplete. Future studies leveraging on lower sequencing costs, technological advances such as application of machine learning for noncoding variant prioritization, ATAC-seq on clinical specimens, single-cell genomics and genome-scale genetic screens, as well as on the wealth of epigenomic datasets that are already available, will further the discovery of diabetes risk mechanisms and aid patient stratification by molecular aetiology.
